# Using a Relational Database to Index Infectious Disease Information

**DOI:** 10.3390/ijerph7052177

**Published:** 2010-05-04

**Authors:** Jay A. Brown

**Affiliations:** Consultant, Specialized Information Services, National Library of Medicine, 6707 Democracy Boulevard, Suite 510, Bethesda, MD 20892, USA; E-Mail: brownjay@haz-map.com

**Keywords:** decision support, relational database, infectious diseases, public health informatics, early detection, differential diagnosis, indexing information, knowledge mapping

## Abstract

Mapping medical knowledge into a relational database became possible with the availability of personal computers and user-friendly database software in the early 1990s. To create a database of medical knowledge, the domain expert works like a mapmaker to first outline the domain and then add the details, starting with the most prominent features. The resulting “intelligent database” can support the decisions of healthcare professionals. The intelligent database described in this article contains profiles of 275 infectious diseases. Users can query the database for all diseases matching one or more specific criteria (symptom, endemic region of the world, or epidemiological factor). Epidemiological factors include sources (patients, water, soil, or animals), routes of entry, and insect vectors. Medical and public health professionals could use such a database as a decision-support software tool.

## 1. Introduction

In the twenty-first century, we now have new tools for indexing infectious disease information (the relational database) and for disseminating that information worldwide (the Internet). A book such as the *Control of Communicable Diseases Manual* has a helpful index, but it does not provide the powerful queries of a relational database, and it cannot be accessed by the millions of computer users around the world, including those in lower-income countries.

A relational database has many features that make it useful as a decision-support tool for health professionals. A relational database can store an unlimited amount of data. Most computer users are familiar with queries, a relatively simple and straightforward method of “zooming-in” on information. An “intelligent database” could be designed to include the most useful information needed for the early diagnosis of infection diseases.

Parsaye and Chignell introduced the concept of intelligent databases as “databases that manage information in a natural way, making information easy to store, access and use” [1]. Designing an intelligent database is similar to the ancient craft of mapmaking. Parsaye and Chignell called this a process of “concentric design” in which the major geographic features (coastlines, major rivers, and mountains) are drawn first. The details are added later, as they are discovered, and in the context of the major features.

Knowledge mapping begins with the big picture, and all of the information is kept in perspective. The need for knowledge mapping is great because there is so much information about infectious diseases; it is easy to get lost in the details. Thousands of new pages of information are published daily in journal articles, book chapters, and Internet websites. The problem today is how to extract the specific information needed and how to make it accessible to people who can intervene and prevent disease [2].

Intelligent action depends upon a good indexing system. One author has written, “Indexing is a major problem at the heart of intelligence. No intelligent system is likely to function effectively if it cannot find what it knows when it needs to know it” [3].

The computer’s strength in data storage and computational speed complements the human strength of general knowledge, creativity, and perspective on the usefulness of the information. To be useful, the large amount of information available must be organized into a format that is easy to query and understand. Otherwise, the quantity of information can be overwhelming and lead to decisions that are wrong or suboptimal. This organizing process involves extracting the critical information from the huge volume of available data [1]. The preliminary work done in sifting through the information and classifying it for digital processing enables users “to focus on the key problems while ignoring a vast sea of irrelevancy” [4].

## 2. Methods

To identify or diagnose infectious diseases, we need the facts at our fingertips. All pertinent infectious disease information was mapped into a relational database using structured and unambiguous indexes. To define the features of this decision-support system, the author explored the world’s scientific literature including PubMed, the websites of CDC and WHO, and book chapters on emerging infectious diseases, bioterrorism, foodborne illnesses, travel-related infections, zoonoses, and other communicable diseases. The system was designed to include these key features:

- Information was comprehensively collected. A content expert, familiar with infectious diseases and clinical diagnosis, gathered the information.
- All information was selected or not selected based on its usefulness for the early detection of infectious diseases.
- Information was systematically indexed by a content expert using hierarchical categories and a controlled vocabulary.

Computer systems have revolutionized the management of medical information. In a fraction of a second, the relational database sorts hundreds of records or returns the results of a query defined by one or more criteria. One of the advantages of a relational database over a flat file database is that records in one table can be related to records in another table. So, a symptom in the Findings table, e.g., eosinophilia, can be named and defined once and then linked to all diseases with that symptom. In an intelligent database, one can store systematized indexes and retrieve matching diseases quickly by simply relying on the built-in power of relational databases to find the records that match the criteria of the query. No complex or hidden algorithms are needed. A query for all disease with the finding “rash” returns one list of diseases. Another query for all diseases with the finding “cough” returns a different list. An AND query is the intersection of these two lists—all disease that have both findings.

Two or three criteria are all you need for a query that will reduce the results list (differential diagnosis) down to manageable size. The following table shows a few examples of important criteria to use in searches. An effective query will combine one or more criteria in one column with one or more criteria in a second column. Add an ENDEMIC criteria (not shown in table) if you want to limit the search to a specific region of the world. OR queries are also possible, but they are not as useful. An example of an OR search is to add all of the countries that a sick traveler recently visited so that all diseases from all countries would be considered in the differential diagnosis.

Putting together a company database is much simpler than developing an intelligent database of infectious diseases. First one must decide which tables to include, e.g., Employees, Customers, Products, Invoices, *etc.* Then add the fields to each table to store the data. So, for the employees table, those fields would include first name, last name, address, city, *etc.* Then link the tables together to show the relationships between tables. The Customers table would be linked to the Invoices table, and each customer could have one or more invoices.

For a relational database of medical information, the job is more complex. One of the most difficult tasks is finding the best indexes to use. As shown by the examples in Table 1, these indexes are the search criteria used to return the differential diagnoses. Thus, for the diseases table the developer must decide which diseases to include and which fields to add. As in a spreadsheet, each row is a disease record, and each column is a field. Some of the information can be displayed in text fields and yes/no fields within the tables and some as links to related tables. The final version of the infectious disease database described here contains 275 diseases and 119 signs & symptoms linked to these diseases. Determining which diseases to include is different for each database depending on the scope and purpose of the database. See the Discussion section for more on the intended audience of this and other databases created by the author.

*Control of Communicable Diseases Manual, 19^th^* *Edition* (CCDM) [5] is a good place to start for which infectious diseases to include and what constitutes the key indexes for separating one disease from another. For each disease, CCDM shows main symptoms, common laboratory tests, place of occurrence, reservoir, incubation period, and other useful data on period of communicability, susceptibility, methods of control, *etc*. But, CCDM does not use a controlled vocabulary of signs and symptoms, and the findings listed for each disease are not applied in a systematic way. It is the job of the intelligent-database developer to create a comprehensive list of findings and to decide for each disease which findings apply. This often requires more research into other sources of information in books, journals, and websites. See the Results section for the main sources of information used.

Building an intelligent database is an iterative process of gathering and refining information. The author started a database of occupational diseases in 1991. Some the occupational diseases were infectious diseases, and the first tables of diseases and findings were begun then. Since then the tables have gone through many revisions to improve the indexes and case definitions.

## 3. Results

The 275 infectious diseases are classified by category, disease type, and acuity (acute-severe, acute-moderate, or subacute). Categories include Arthropod-Borne, Biological Weapons, Childhood Infections, Community-Acquired, Foodborne, Gastroenteritis, Localized Infections, Sapronoses, Sexually-Transmitted, and Zoonoses. Disease types are Bacteria, Fungus, Helminth, Mixed, Other, Protozoa, Rickettsia, Spirochete, Toxin, and Virus. The database can be filtered to show only one category, one disease type, or one level of acuity. For each disease, the application shows initial symptoms, incubation period, signs and symptoms, and any activities associated with increasing the risk of disease. There are 119 signs and symptoms in thirteen categories as shown in Table 2. The application shows where the disease occurs in the world; how it is diagnosed in the laboratory; its source from patients, water, soil, and animal excreta or tissue; its route of entry into the body by inhalation, ingestion, or skin; and the insect vectors and animal reservoirs.

**Figure 2.**
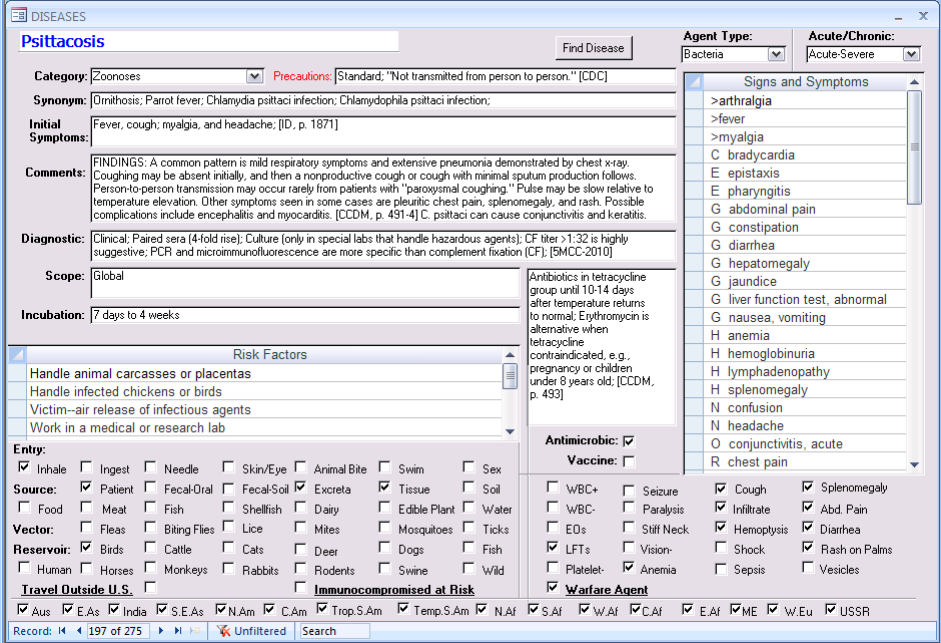
Psittacosis is one of the 275 infectious diseases shown here in the Microsoft Access user interface in which the application was developed and continues to be updated.

As shown in Figure 2, the three main tables in the database are Diseases, Findings (signs & symptoms), and High-Risk Activities. Each disease is also linked to one or more of 16 specific regions of the world in which it is endemic, and to epidemiological factors. These links to regions and factors are made, not by linking to tables, but by additional fields within the Diseases table. So, for example, every disease carried by ticks has the “yes” selected in the yes/no field called “Di_Ticks” in the Diseases table. Most of the 79 fields in the Diseases table are yes/no fields, but some are text fields that capture, for example, the disease names or the incubation periods. Yes/no fields in the diseases table serve as criteria that can be used in a query, e.g., show all diseases that have the tick field checked. In the application described, the incubation period is a text field, and it cannot be used in a query. However, it would be easy to add this feature by simply adding several yes/no fields for incubation periods of hours, days, weeks, months, and years. Then, a query for all diseases with an incubation period of months could be returned in a query.

Having information organized into a relational database means that the user can find diseases by means of queries. For example, the user can query the database of 275 diseases to find all that match certain criteria:

- A sign or symptom, e.g., cough, paralysis, bradycardia, neutropenia, petechiae, or pleural effusion;
- An activity, e.g., have a blood transfusion, drank unpasteurized milk, or took care of a sick bird; or

The user can also perform “AND” searches, for example, all the diseases that match:

- Travel to central Africa AND bleeding diathesis;
- Cat contact AND diarrhea;
- Tick exposure AND decreased platelets;

Information from textbooks [5–16], journal articles [17–26], online resources [27–30], and electronic databases [31–34] was classified and summarized to create the database. A bibliography including online resources is regularly updated on the author’s website [35]. This web page also shows the reference labels that are used throughout the application to reference source material within the content of the database, e.g., “CCDM” [5] and “PPID” [6]. References to resources used in Haz-Map (toxicology and occupational medicine) can be viewed from the “References” tab at the Haz-Map website [36].

The profile for the disease Psittacosis is shown in Figure 3. The form displays the database fields of information, e.g., the incubation period, and it shows the linked findings and high-risk activities. Psittacosis is present in all regions of the world as shown in the bottom row of checkmarks. Above this on the left are the Epidemiological Factors. Risk Factors (high-risk activities) and Findings are displayed as subforms within the main form. Each finding and risk factor is a controlled vocabulary term or index that is being used to display information about the disease in this form. The same controlled vocabulary terms are used in queries.

The list of findings is sorted by prefixes to make it more readable, *i.e.*, so that signs & symptoms for each organ system are grouped together. The prefixes are defined in Table 2. General symptoms such as fever, fatigue, and myalgia are not very useful in distinguishing one infectious disease from another, but they are still important to show for each disease. In the infectious disease database currently being described, 30 of the 119 findings are complications. A complication such as encephalitis is important to show for each disease even though it may complicate the infectious disease in only one percent of cases, or even less.

Each disease also has a field for “Initial Symptoms” to summarize the first symptoms experienced by the majority of patients. When the frequency of symptoms is known from one or more case series, then this is documented in the Comments field. The references are also shown in the Comments field to document the sources of information for each linked finding and epidemiological factor. If there is contradictory information from two sources, then one or more additional sources are consulted.

Developing an intelligent database of infectious diseases has some similarities with editing an infectious diseases textbook. The work requires the collection of evidence and the summarization of the current state of knowledge. The editor uses his best judgment in deciding which diseases to include and how much to say about each one. Some symptoms will be emphasized because they are common, and some will not be mentioned because they have only been described in one case. Also, the editor relies on the latest reports and summaries from the scientific literature to update the profile of each disease. The case definitions are never finished because they are in the ever-refining, iterative loop of the surveillance cycle as shown in Table 3.

## 4. Discussion

### 4.1. The Value of Intelligent Databases

Using two screening tests in a series increases the positive predictive value (PPV) compared to a single test [37]. When a practitioner takes a patient’s medical history with access to an accurate differential diagnosis, this is like performing a screening test. It precedes laboratory studies and should decrease the number of costly lab tests needed. Increasing the PPV in this way helps to address a major roadblock to identifying and preventing infectious diseases, especially in developing countries. To be effective, increasingly sensitive diagnostic tests must be accompanied by increasingly well-trained health professionals who can “make clinical sense of the results” [38].

### 4.2. The Limitations of Intelligent Databases as Decision-Support Systems

Having a software tool is not the same as having an infectious disease specialist with years of training and experience diagnosing infectious diseases. An intelligent database is like an electronic book that you can query. It is not designed to replace the human mind, but to assist the professional with lists and intersections of lists. For example, one can make a list of all infections that cause abdominal pain and another list of all infections caused by animal bites. The intersection of those two lists is all infections that fit both criteria. The physicians who make these databases must first have good comprehension of the domain and then do the research necessary to define the best criteria (indexes) that will return the most useful lists of differential diagnoses. Unlike books, in which the index items are added last, the indexes in an intelligent database are established at the beginning of the project. Each index is a controlled vocabulary term that is used to query the database. The database is only as good as the research that the database developer has done to determine the best indexes and to accurately link each disease with all indexes that apply. Thus, for “eosinophilia,” the medical scientist must determine for each disease whether or not it is associated with this finding.

Some artificial intelligence systems are designed to act like replacements of the astute clinician. The user types in the case findings, and then the system comes up with the answer. For intelligent database systems, the emphasis is more on assistance than replacement. What kinds of information can the computer store that will help the clinician solve the problem? As shown in the examples in Table 1, there are a number of key criteria that can be used in queries to build and shorten the differential diagnosis list. Since the computing process that builds the list is completely transparent, the health professional can consider the results of the software tool that may or may not help with a particular case. As the tool gets more accurate with time and the professional learns better the limitations of the tool, then there will be more cases in which the software can make a difference in terms of earlier and more accurate diagnoses.

Some infectious disease cases are difficult to diagnose. Signs and symptoms may be misinterpreted, mixed up, or just missed. Therefore, the differential diagnosis may be wrong from the beginning because the signs and symptoms of the case have not been correctly identified. Here are some examples. An ophthalmologist misnamed a case of optic neuritis as papilledema. A patient was found to have scleral icterus by the emergency room doctor, but the admitting labs showed normal serum bilirubin. A rash may be called maculopapular, vesicular, and pustular by different observers at different times. A rash may be caused by a complication of the disease, e.g., Stevens Johnson syndrome, rather than by the disease itself. More than one disease may be causing the signs and symptoms. The disease may be non-infectious such as a metastatic tumor or plaque emboli. Insignificant findings may be magnified while important ones are ignored. Because of these difficulties, the judgment of an alert and well-trained medical professional is critical to making a correct diagnosis with or without decision-support software.

### 4.3. The Potential Uses of Intelligent Databases

An Internet interface for the intelligent database described in this article could be developed within a few months. A PDA (personal digital assistant) interface has already been developed. Haz-Map®, the author’s first intelligent database, was first published on the US National Library of Medicine website in 2002 with free database access [39]. Although primarily a database of toxic chemicals, Haz-Map also includes occupational infections. An intelligent database of outbreaks that covers all diseases known to cause acute syndromes in clusters of patients has also been developed. That database includes mainly infectious diseases, but also those caused by chemical accidents and bioterrorist attacks. Both Haz-Map and the outbreaks database provide the user with the ability to do queries by occupation.

The threat of bioterrorism has a silver lining in that it brings our attention to the need for better education of health professionals regarding emerging infectious diseases. There is a need for surveillance systems that can detect outbreaks more rapidly, and these systems will depend on the active participation of front-line physicians [40]. Bioterrorism diseases would likely present as other common diagnoses in primary care. Educating family physicians to recognize bioterrorism events is of the highest priority [41]. Properly designed decision-support systems could help clinicians to identify bioterrorist events by generating lists of differential diagnoses given the clinical features of the case [42]. Our efforts to respond to bioterrorism attacks are useful to society even if a terrorist attack never occurs. We will always be exposed to outbreaks from nature; and so the more we learn about how to respond to bioterrorism, the better prepared we will be in the event of any outbreak [43,44].

An Internet version of this infectious disease database may be useful for clinical and public health professionals in developing countries. Because of its simplicity, the need for training should be minimal. In detecting and responding to infectious diseases in lower-income countries, “the empowerment of frontline health workers and communities is a key element for an efficient surveillance system” [45].

### 4.4. Other Decision-Support Systems Already on the Internet

There are several resources already available on the Internet to help professionals around the world diagnose infectious diseases. The electronic version of *The Control of Communicable Diseases Manual* was first published in March 2010. A one-year subscription for access to both the web-based and PDA software costs $49.95 [46]. This is an electronic book that can be searched, but not a relational database that can be queried by predetermined indexes.

The Johns Hopkins *ABX Guide* is a free website designed to help clinicians by distilling and updating complex information about infectious diseases and their treatment [47]. The user can drill down to diagnosis categories including biodefense, neurologic, respiratory, fever, and travel. The user can also go to categories of antimicrobial drugs and to types of pathogens.

“Practice Guidelines for Evaluation of Fever in Returning Travelers or Migrants” is another free website for health professionals to provide a differential diagnosis based on the countries visited, incubation period, high-risk activities, signs, symptoms, and laboratory results [48]. The guidelines were published in a scientific paper with the same name after a systematic review of the literature [49]. The algorithm was designed to be used for the first evaluation of patients who are not immunocompromised, pregnant, children, or suffering from any underlying chronic diseases. It is restricted to tropical and subtropical diseases. The authors note that, “No estimation of the relative probability of each diagnosis in a given situation has been mentioned since no good incidence data exist.”

Finally, GIDEON (Global Infectious Disease and Epidemiology Network) was first designed in 1990 using Paradox, one of the first DOS-based relational databases. GIDEON is now available as a web-based application for $995 per year [50]. In GIDEON, “The differential diagnosis list is generated by a Bayesian formula which compares the product of disease-incidence and symptoms incidence, for all compatible infectious diseases” [51].

In a study of hospitalized patients in Boston, GIDEON found the correct diagnosis in 69% of the cases and listed it first in 60% of the cases [52]. The author has noted the problem with treating individual countries as whole units with regard to which diseases are considered endemic; he has also admitted the problem with “the availability and quality of valid epidemiological data” [51]. A Japanese study of 98 febrile travelers found that the correct diagnosis appeared in GIDEON’s differential diagnosis list in 91% of the cases [53].

A more recent evaluation of GIDEON found that the software generated a median of 29 diagnoses per case [54]. Of the 129 cases of fever in returning travelers or migrants, the correct diagnosis was generated by GIDEON in the top 5 ranking for 64% of the febrile episodes. The authors of this study noted the problem of nonspecific symptoms, e.g., myalgia, being variably listed when describing a typical disease presentation. They also warned that using the software “might be hazardous for inexperienced physicians.” (Of course, medical textbooks might be hazardous for inexperienced physicians as well.) The authors point out the trend since the 1980s for decisions-support systems to be less like Greek oracles reciting the truth to passive physicians and more like tools that actively thinking physicians use, all the while aware of their limitations. In the study of hospitalized patients in Boston, the authors noted that GIDEON correctly supplied a leading diagnosis not considered by the admitting team in five cases. This is a quality of a useful tool—that it can help the worker do the job better with the tool than without it.

## 5. Conclusions

A new research opportunity came into being with the availability of user-friendly relational databases in the early 1990s. A wealth of information about infectious diseases exists in journals of emerging infectious diseases and textbooks of internal, tropical, military, and occupational medicine, but “Information without access to that information is no information at all” [3]. It is time to collect all of this information, classify it, index it, and store it in one computer database for continuous refining and updating. By providing quick access to the facts, such a relational database could help medical and public health professionals to actively participate in the surveillance cycle and to diagnose infectious diseases earlier and more accurately.

## Figures and Tables

**Figure 1.**
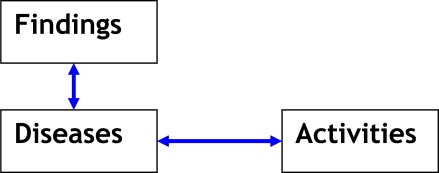
Bidirectional arrows represent many-to-many relationships between tables, meaning that one can see all diseases linked to a finding and all findings linked to a disease.

**Table 1.** Some Examples of Search Criteria.

FINDINGS	ENTRY	VECTOR	RESERVOIR
Abdominal pain	Inhale	Biting Flies	Birds
Encephalitis	Ingest	Fleas	Cats
Pneumonia	Animal bite	Lice	Cattle
Jaundice	Needle	Mosquitoes	Dogs
Stiff Neck	Sexual	Ticks	Fish

**Table 2.** Finding Categories and the Prefixes Used for Abbreviations.

**Prefix**	**Finding Category**
>	General
C	Cardiovascular
E	Ears, Nose & Throat,
G	Gastrointestinal
H	Hematologic
M	Musculoskeletal
N	Neurologic
O	Ophthalmologic
R	Respiratory
S	Skin
U	Genitourinary
X	Chest X-ray
*	Complication

**Table 3.**
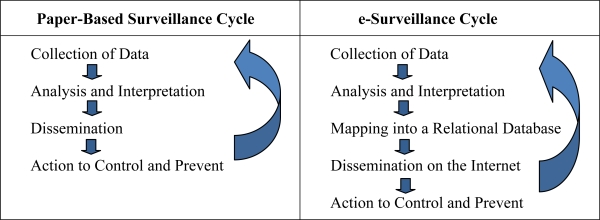
Modification of the surveillance cycle that is possible today in the age of electronic databases and the Internet.

## References

[1] Parsaye K Chignell M Intelligent Database Tools & Applications John Wiley & Sons New York, NY, USA 1993 453512

[2] Berner ES Moss J Informatics challenges for the impending patient information explosion J. Am. Med. Inform. Assoc 2005 12 614 617 16049224 10.1197/jamia.M1873 PMC1294032

[3] Schank RC Tell Me a Story: Narrative and Intelligence Northwestern University Press Evanston, IL, USA 1995 11112 113

[4] O’Carroll PW Yasnoff WA Ward ME Ripp LH Martin EL Public Health Informatics and Information Systems Springer New York, NY, USA 2003 18496

[5] Heymann DL Control of Communicable Diseases Manual19th APHA Washington, DC, USA 2008

[6] Mandell GL Bennett JE Dolin R Principles and Practice of Infectious Diseases7th ed Elsevier Philadelphia, PA, USA 2010

[7] Gorbach SL Bartlett JG Blacklow NR Infectious Diseases3rd ed Lippincott Williams & Wilkins Philadelphia, PA, USA 2004

[8] Beers MH Porter RS Jones T Kaplan JL Berkwits M The Merck Manual18th Merck & Co., Inc. Whitehouse Station, NJ, USA 2006

[9] Goldman L Ausiello D Cecil Medicine23rd ed Saunders Elsevier Philadelphia, PA, USA 2008 2103

[10] Guerrant RL Walker DH Weller PF Tropical Infectious Diseases2nd ed Churchill Livingston Philadelphia, PA, USA 2006

[11] Wallace RB Maxcy-Rosenau-Last Public Health & Preventive Medicine15th ed McGraw-Hill New York, NY, USA 2008 55

[12] Wallach J Interpretation of Diagnostic Tests8th ed Lippincott Williams & Wilkins Boston, MA, USA 2007

[13] Brunette GW Kozarsky PE Magill AJ Shlim DR Whatley AD CDC Health Information for International Travel 2010 Mosby Elsevier Philadelphia, PA, USA 2009

[14] U.S. Army Medical Research Institute of Infectious Diseases USAMRIID Medical Management of Biological Casualties Handbook, 6th edAvailable online: http://www.usamriid.army.mil/education/bluebookpdf/USAMRIID%20BlueBook%206th%20Edition%20-%20Sep%202006.pdf (accessed on December 13, 2009).

[15] Weinstein RS Alibeck K Biological and Chemical Terrorism Thieme New York, NY, USA 2003

[16] Isada CM Kasten BL Goldman MP Gray LD Aberg JA Lexi-Comp’s Infectious Diseases Handbook5th ed. Lexi-Comp Hudson, OH, USA 2003

[17] Borio L Inglesby T Peters CJ Schmaljohn AL Hughes JM Jahrling PB Ksiazek T Johnson KM Meyerhoff A O’Toole T Ascher MS Bartlett J Breman JG Eitzen EM Jr Hamburg M Hauer J Henderson DA Johnson RT Kwik G Layton M Lillibridge S Nabel GJ Osterholm MT Perl TM Russell P Tonat K Hemorrhagic fever viruses as biological weapons: medical and public health management JAMA 2002 287 2391 2405 11988060 10.1001/jama.287.18.2391

[18] Dennis DT Inglesby TV Henderson DA Bartlett JG Ascher MS Eitzen E Fine AD Friedlander AM Hauer J Layton M Lillibridge SR McDade JE Osterholm MT O’Toole T Parker G Perl TM Russell PK Tonat K Tularemia as a biological weapon: medical and public health management JAMA 2001 285 2763 2773 11386933 10.1001/jama.285.21.2763

[19] Henderson DA Inglesby TV Bartlett JG Ascher MS Eitzen E Jahrling PB Hauer J Layton M McDade J Osterholm MT O’Toole T Parker G Perl T Russell PK Tonat K Smallpox as a biological weapon: medical and public health management. Working Group on Civilian Biodefense JAMA 1999 281 2127 2137 10367824 10.1001/jama.281.22.2127

[20] Henretig FM Cieslak TJ Kortepeter MG Fleisher GR Medical management of the suspected victim of bioterrorism: an algorithmic approach to the undifferentiated patient Emerg. Med. Clin. North Am 2002 20 351 364 12120483 10.1016/s0733-8627(01)00005-0

[21] Hubalek Z Emerging human infectious diseases: anthroponoses, zoonoses, and sapronoses Emerg. Infect. Dis 2003 9 403 404 12643844 10.3201/eid0903.020208 PMC2958532

[22] Inglesby TV Dennis DT Henderson DA Bartlett JG Ascher MS Eitzen E Fine AD Friedlander AM Hauer J Koerner JF Layton M McDade J Osterholm MT O’Toole T Parker G Perl TM Russell PK Schoch-Spana M Tonat K Plague as a biological weapon: medical and public health management. Working Group on Civilian Biodefense JAMA 2000 283 2281 2290 10807389 10.1001/jama.283.17.2281

[23] Inglesby TV O’Toole T Henderson DA Bartlett JG Ascher MS Eitzen E Friedlander AM Gerberding J Hauer J Hughes J McDade J Osterholm MT Parker G Perl TM Russell PK Tonat K Anthrax as a biological weapon, 2002: updated recommendations for management JAMA 2002 287 2236 2252 11980524 10.1001/jama.287.17.2236

[24] O’Brien KK Higdon ML Halverson JJ Recognition and management of bioterrorism infections Am. Fam. Physician 2003 67 1927 1934 12751654

[25] Reingold AL Outbreak investigations—a perspective Emerg. Infect. Dis 1998 4 21 27 9452395 10.3201/eid0401.980104 PMC2627658

[26] Arnon SS Schechter R Inglesby TV Henderson DA Bartlett JG Ascher MS Eitzen E Fine AD Hauer J Layton M Lillibridge S Osterholm MT O’Toole T Parker G Perl TM Russell PK Swerdlow DL Tonat K Botulinum toxin as a biological weapon: medical and public health management JAMA 2001 285 1059 1070 11209178 10.1001/jama.285.8.1059

[27] Centers for Control and Prevention (CDC) Guidelines for Infection Control in Health Care Personnel. Available online: http://www.cdc.gov/ncidod/dhqp/gl_hcpersonnel.html (accessed on December 13, 2009).

[28] Centers for Disease Control and Prevention (CDC) Biosafety in Microbiological and Biomedical Laboratories (BMBL). Available online: http://www.cdc.gov/od/ohs/biosfty/bmbl4/bmbl4toc.htm (accessed on December 13, 2009).

[29] Centers for Disease Control and Prevention (CDC) Diagnosis and Management of Foodborne Illnesses. MMWR Vol. 53/RR-4 Available online: http://www.cdc.gov/mmwr/PDF/rr/rr5304.pdf (accessed on December 13, 2009).

[30] Centers for Disease Control and Prevention(CDC) Guideline for Isolation Precautions: Preventing Transmission of Infectious Agents in Healthcare Settings. Appendix A. Available online: http://www.cdc.gov/ncidod/dhqp/gl_isolation_appendixA.html (accessed on December 13, 2009).

[31] Bartlett JG Auwaeter PG Pham P ABX GuideAvailable online: http://prod.hopkins-abxguide.org/ (accessed on December 13, 2009).

[32] General Hospital Corporation DXplain: Diagnostic Decision Support. Available online: http://www.merckmedicus.com/pp/us/hcp/frame_dxplain.jsp?pg=www.merckmedicus.com/pp/us/hcp/dxplain_home.jsp (accessed on December 13, 2009).

[33] Domino FJ Baldor RA Golding J Grimes JA Taylor JS The 5-Minute Clinical Consult 2010Available online: http://www.5mcc.com (accessed on December 13, 2009).

[34] Fauci AS Braunwald E Kasper DL Hauser SL Longo DL Jameson JL Loscalzo J Harrison’s Practice—Answers on DemandAvailable online: http://www.merckmedicus.com/ (accessed on December 13, 2009).

[35] Brown JA OutbreakID: BibliographyAvailable online: http://outbreakid.com/bibliography.htm (accessed on December 13, 2009).

[36] U.S. National Library of Medicine Haz-Map: Occupational Exposure to Hazardous Agents. Available online: http://hazmap.nlm.nih.gov/ (accessed on December 13, 2009).

[37] Hennekens CH Buring SL Epidemiology in Medicine Little, Brown and Company Boston, MA, USA 1987

[38] Smolinski MS Hamburg MA Lederberg J Microbial Threats to Health: Emergence, Detection, and Response The National Academies Press Washington, DC, USA 2003 xvii180 25057653

[39] Brown JA An internet database for the classification and dissemination of information about hazardous chemicals and occupational diseases Am. J. Ind. Med 2008 51 428 435 18335440 10.1002/ajim.20578

[40] Nierengarten MB Lutwick L Lutwick S Syndrome-Based Surveillance for Clinicians on the Frontlines of Healthcare: Focus on Rapid Diagnosis and NotificationAvailable online: http://cme.medscape.com/viewprogram/2427 (accessed on December 13, 2009).

[41] Temte JL Zinkel AR The primary care differential diagnosis of inhalational anthrax Ann. Fam. Med 2004 2 438 444 15506578 10.1370/afm.125 PMC1466714

[42] Bravata DM Sundaram V McDonald KM Smith WM Szeto H Schleinitz MD Owens DK Evaluating detection and diagnostic decision support systems for bioterrorism response Emerg. Infect. Dis 2004 10 100 108 15078604 10.3201/eid1001.030243 PMC3322751

[43] Eitzen EM Jr Education is the key to defense against bioterrorism Ann. Emerg. Med 1999 34 221 223 10424925 10.1016/s0196-0644(99)70233-7

[44] Rosen P Coping with bioterrorism is difficult, but may help us respond to new epidemics BMJ 2000 320 71 72 10625245 10.1136/bmj.320.7227.71 PMC1117387

[45] Calain P From the field side of the binoculars: a different view on global public health surveillance Health Policy Plan 2007 22 13 20 17237490 10.1093/heapol/czl035

[46] Control of Communicable Diseases Manual for Mobile + Web. Available online: http://www.unboundmedicine.com/store/communicable_diseases (accessed on April 20, 2010).

[47] ABX ABX GuideAvailable online: http://hopkins-abxguide.org/ (accessed on April 20, 2010).

[48] Practice Guidelines for Evaluation of Fever in returning Travelers or Migrants. Available online: http://www.fevertravel.ch/ (accessed on April 20, 2010).

[49] D’Acremont V Ambresin AE Burnand B Genton B Practice guidelines for evaluation of Fever in returning travelers and migrants J. Travel Med 2003 10 S25 S52 12740187 10.2310/7060.2003.35132

[50] Global Infectious Disease and Epidemiology NetworkAvailable online: http://www.gideononline.com/ (accessed on April 10, 2010).

[51] Berger SA GIDEON: a comprehensive Web-based resource for geographic medicine Int. J. Health Geogr 2005 4 10 15847698 10.1186/1476-072X-4-10 PMC1090610

[52] Ross JJ Shapiro DS Evaluation of the computer program GIDEON (Global Infectious Disease and Epidemiology Network) for the diagnosis of fever in patients admitted to a medical service Clin. Infect. Dis 1998 26 766 767 9524864 10.1086/517123

[53] Kimura M Sakamoto M Adachi T Sagara H Diagnosis of febrile illnesses in returned travelers using the PC software GIDEON Travel Med. Infect. Dis 2005 3 157 160 17292033 10.1016/j.tmaid.2004.08.003

[54] Bottieau E Moreira J Clerinx J Colebunders R Van Gompel A Van den Ende J Evaluation of the GIDEON expert computer program for the diagnosis of imported febrile illnesses Med. Decis. Making 2008 28 435 442 18310530 10.1177/0272989X07312715

